# Characterization of the mitochondrial genome of *Tinda javana* (Macquart, 1838) (Diptera: Stratiomyidae: Pachygastrinae)

**DOI:** 10.1080/23802359.2021.1910081

**Published:** 2021-04-15

**Authors:** Kai Hu, Zaihua Yang

**Affiliations:** Guizhou Academy of Forestry, Guiyang, PR China

**Keywords:** Mitogenome, phylogenetic analysis, Brachycera, Pachygastrinae, Tinda

## Abstract

We have assembled and annotated the mitochondrial genome of *Tinda javana* (Macquart, 1838) (a species of soldier fly) in this study. It is 15,495 bp in length, including 13 protein-coding genes (PCGs), two ribosomal *RNA* genes (*rRNA*s), 22 transfer *RNA* genes (*tRNA*s), and a large non-coding control region (length: 704 bp). The nucleotide composition of whole mitochondrial genome biases toward A and T (75.5%). Most PCGs use ATN as initiation codon, except for *cox1* which starts with CGA. All PCGs end with common termination codon TAA/G. Phylogenetic analyses based on the nucleotide sequence data supported the monophyly of Stratiomyidae and the sister relationship between Pachygastrinae and the clade (Nemotelinae + (Hermetiinae + Sarginae)).

Pachygastrinae is a subfamily of flies, which belongs to the family Stratiomyidae of Diptera. It is a larger subfamily in Stratiomyidae and widely distributes all over the world. Up to now, 619 species of 180 genera are known worldwide (Woodley 2001; Yang, Zhang, et al. 2014). Larvae of some species feed on *Musa* L. plants and are important agricultural and forestry pests (Yang ZH, Yang Y, et al. 2014). Classification and systematics of Pachygastrinae are of great interest for management of plant pests. This study firstly sequenced the mitochondrial genome of *Tinda javana* to discuss the phylogenetic status of Pachygastrinae.

The samples of *T. javana* were collected from Luodian County (E106.7917, N25.4075), Guizhou, China, and deposited in Insect Herbarium of Guizhou Academy of Forestry, Guiyang (GZAF-2020-DS1445) (URL, Zaihua Yang and yangzaihua008@126.com). The total genome DNA was sequenced using Illumina MiSeq format (Illumina, San Diego, CA). Then the data was assembled by NOVOPlasty version 2.7.0 (Dierckxsens et al. 2017) with the *cox1* gene of *Nemotelus notatus* (MT584142) as the seed. The complete mitochondrial genome was annotated using MITOZ version 1.04 (Meng et al. 2019). All 37 mitochondrial gene sequences were aligned using MAFFT version 7.394 (Kuraku et al. 2013) with G-INS-I (accurate) strategy. Maximum likelihood (ML) tree was inferred by IQ-TREE version 1.6.3 (Nguyen et al. 2015) under the optimal model (GTR + I + G for Subset1 (*nad3*, *atp6*, *cox1*, *cox2*, *cytb*, and *cox3*), Subset2 (*atp8*, *nad2*, and *nad6*), and Subset3 (*nad1*, *nad4L*, *nad4*, and *nad5*); TVM + G for Subset4 (*rrnL* and *rrnS*); TVM + I + G for Subset5 (*trnM*, *trnW*, *trnK*, *trnV*, *trnC*, *trnI*, *trnP*, *trnH*, *trnE*, *trnD*, *trnT*, *trnL1*, *trnL2*, *trnN*, *trnG*, *trnS2*, *trnR*, *trnA*, and *trnS1*); HKY + G for Subset6 (*trnQ*, *trnY*, and *trnF*)).

The complete mitogenome of *T. javana* (Genbank accession no. MW115422) is 15,495 bp in length with highly AT biased of nucleotide composition (38.1% A, 37.4% T, 9.9% G, and 14.6% C). All 37 typical mitochondrial genes were annotated in this newly sequenced species, containing 13 PCGs, 22 *tRNA*s, two *rRNA*s, and control region. The mitochondrial genome of *T. javana* shares the same gene order as ancestral insects such as *Drosophila yakuba* and *Drosophila mercatorum* (Clary and Wolstenholme 1985; Wang et al. 2019). Fifteen tRNAs and nine PCGs are encoded on the majority strand (J-strand) while the remaining genes are encoded on the minority strand (N-strand). A total of ten overlaps (1–8 bp) between adjacent genes were found, the longest overlap is 8 bp between *trnW* and *trnC*. The intergenic spacers were detected in 12 locations, ranging from 1 to 18 bp. The length of 13 PCGs is 11,154 bp, accounting for 72.06% of whole mitochondrial genome. Most PCGs initiated by standard start codon ATN (ATA/T/G/C), except for *cox1* which starts with CGA. All PCGs end with common termination codon TAA/G.

Here, based on the nucleotide sequence data of 37 genes from *T. javana* and other 16 species belonging to seven related families of Diptera, we reconstructed the phylogenetic tree (Figure 1). The ML analyses strongly supported the monophyly of Stratiomyidae (BS = 100), consistent with some previous studies (Zhou et al. 2017; Ding and Yang 2020). In Stratiomyidae, the relationships among included subfamilies are inferred as (Pachygastrinae (*T. javana*) + (Nemotelinae (*Nemotelus notatus*) + (Hermetiinae (*Hermetia illucens*) + Sarginae (*Ptecticus aurifer*)))).

**Figure 1.**
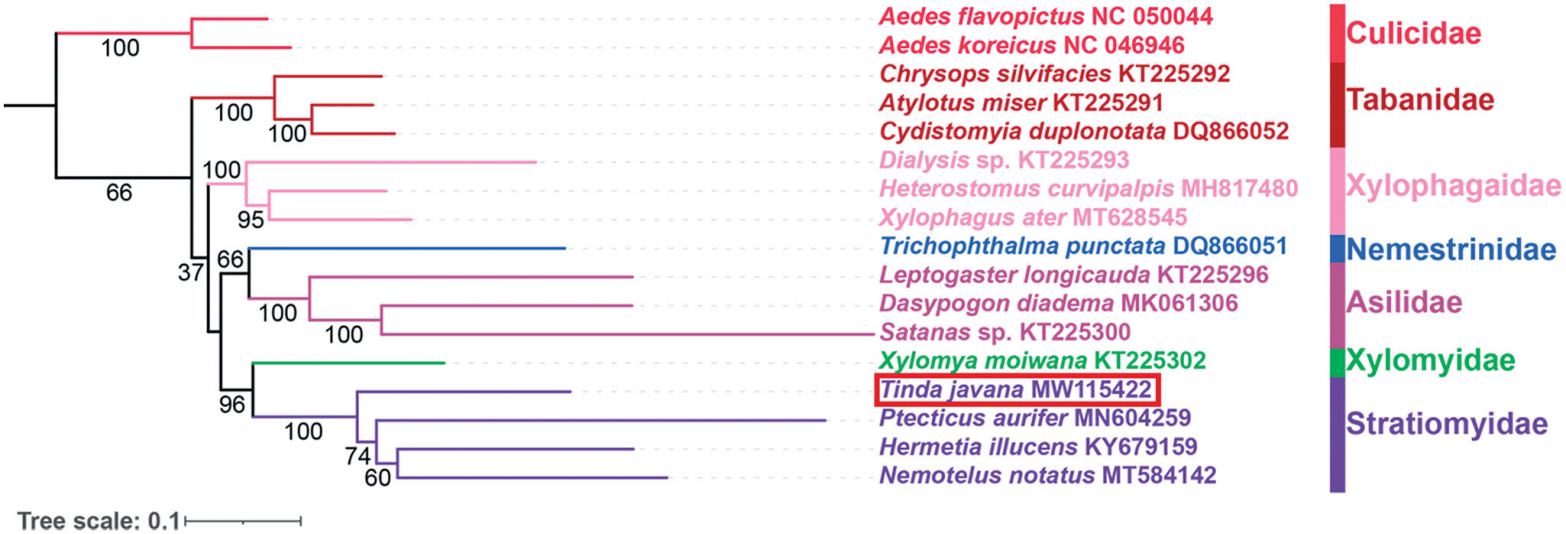
Maximum-likelihood phylogenetic tree based on the nucleotide sequence data of 37 mitochondrial genes from *T. javana* and other 16 species belonging to seven related families of Diptera. The number on each node indicates bootstrap support value.

## Data Availability

The genome sequence data that support the findings of this study are openly available in GenBank of NCBI at (https://www.ncbi.nlm.nih.gov/) under the accession no. MW115422. The associated BioProject, SRA, and Bio-Sample numbers are PRJNA705515, SRR13810270, and SAMN18091015, respectively.
